# A Web-Based Intervention to Reduce Distress After Prostate Cancer Treatment: Development and Feasibility of the Getting Down to Coping Program in Two Different Clinical Settings

**DOI:** 10.2196/cancer.8918

**Published:** 2018-04-30

**Authors:** Jane Cockle-Hearne, Deborah Barnett, James Hicks, Mhairi Simpson, Isabel White, Sara Faithfull

**Affiliations:** ^1^ School of Health Sciences Faculty of Health and Medical Sciences University of Surrey Guildford United Kingdom; ^2^ Time to Talk Brighton General Hospital Sussex Community NHS Foundation Trust Brighton United Kingdom; ^3^ St. Richard's Hospital Western Sussex Hospitals NHS Trust Chichester United Kingdom; ^4^ Access Division - Cancer NHS Lanarkshire Monklands Hospital Airdrie United Kingdom; ^5^ Applied Health Research Group Department of Psychological Support and Pastoral Care Royal Marsden NHS Foundation Trust London United Kingdom

**Keywords:** prostatic neoplasms, Internet, self-management, cognitive behavior therapy, primary health care, secondary care

## Abstract

**Background:**

Distress after prostate cancer treatment is a substantial burden for up to one-third of men diagnosed. Physical and emotional symptoms and health service use can intensify, yet men are reticent to accept support. To provide accessible support that can be cost effectively integrated into care pathways, we developed a unique, Web-based, self-guided, cognitive-behavior program incorporating filmed and interactive peer support.

**Objective:**

To assess feasibility of the intervention among men experiencing distress after prostate cancer treatment. Demand, acceptability, change in distress and self-efficacy, and challenges for implementation in clinical practice were measured.

**Methods:**

A pre-post, within-participant comparison, mixed-methods research design was followed. Phase I and II were conducted in primary care psychological service and secondary care cancer service, respectively. Men received clinician-generated postal invitations: phase I, 432 men diagnosed <5 years; phase II, 606 men diagnosed <3.5 years. Consent was Web-based. Men with mild and moderate distress were enrolled. Web-based assessment included demographic, disease, treatment characteristics; distress (General Health Questionnaire-28); depression (Patient Health Questionnaire-9); anxiety (General Anxiety Disorder Scale-7); self-efficacy (Self-Efficacy for Symptom Control Inventory); satisfaction (author-generated, Likert-type questionnaire). Uptake and adherence were assessed with reference to the persuasive systems design model. Telephone interviews explored participant experience (phase II, n=10); interviews with health care professionals (n=3) explored implementation issues.

**Results:**

A total of 135 men consented (phase I, 61/432, 14.1%; phase II, 74/606, 12.2%); from 96 eligible men screened for distress, 32% (30/96) entered the intervention (phase I, n=10; phase II, n=20). Twenty-four completed the Web-based program and assessments (phase I, n=8; phase II, n=16). Adherence for phase I and II was module completion rate 63% (mean 2.5, SD 1.9) versus 92% (mean 3.7, SD 1.0); rate of completing cognitive behavior therapy exercises 77% (mean 16.1, SD 6.2) versus 88% (mean 18.6, SD 3.9). Chat room activity occurred among 63% (5/8) and 75% (12/16) of men, respectively. In phase I, 75% (6/8) of men viewed all the films; in phase II, the total number of unique views weekly was 16, 11, 11, and 10, respectively. The phase II mood diary was completed by 100% (16/16) of men. Satisfaction was high for the program and films. Limited efficacy testing indicated improvement in distress baseline to post intervention: phase I, *P*=.03, *r*=−.55; phase II, *P*=.001, *r*=−.59. Self-efficacy improved for coping *P*=.02, *r*=−.41. Service assessment confirmed ease of assimilation into clinical practice and clarified health care practitioner roles.

**Conclusions:**

The Web-based program is acceptable and innovative in clinical practice. It was endorsed by patients and has potential to positively impact the experience of men with distress after prostate cancer treatment. It can potentially be delivered in a stepped model of psychological support in primary or secondary care. Feasibility evidence is compelling, supporting further evaluative research to determine clinical and cost effectiveness.

## Introduction

### Need for Psychological Support

In developed regions of the world, men are more likely to be diagnosed with prostate cancer than any other cancer, and those diagnosed are more likely to develop distress or serious psychological problems than healthy men [1,2]. Over 60% of men with prostate cancer report unmet psychological needs and up to a third experience pronounced clinical distress [3-7]. They also have a higher risk of suicide than their healthy male counterparts [8]. A range of factors contribute to men’s psychological comorbidity. Side effects of treatment such as urinary, sexual, bowel, and body-image problems can have a negative effect on cancer-related distress for as much as 2-3 years after diagnosis [9-11], and men’s psychological well-being can be adversely affected by lack of support, the threat of cancer, and the perceived loss of masculine identity [12-14].

The numbers of men with prostate cancer living with and beyond diagnosis are predicted to grow. There are over 1.1 million new prostate cancer cases globally per year, accounting for some 15% of all cancer diagnoses in men [2]. Incidence varies but trends indicate increasing diagnoses and decreasing mortality rates, particularly in developed countries and where screening programs have been implemented [15]. Five-year survival rates now exceed 84% in Western Europe and approach 100% in the United States and Australia [16-18], and in the United Kingdom for instance, incidence rates are expected to rise by 12%, to over 77,000 new cases per year by 2035 [19]. The growing number surviving prostate cancer means there will be more men experiencing reduced psychological well-being and quality of life, resulting in increased care utilization and health service costs. Innovative, accessible, and low-cost care delivery solutions are required to meet this long-term challenge.

Providing early psychological support is vital to ensure men experiencing distress after prostate cancer treatment do not fall into a cycle of negative thinking and avoidance behaviors, which can escalate symptoms and lead to the need for more intensive, prolonged support [5,6]. However, men’s engagement with psychological support is frequently restrained: reticence to communicate and delays in presenting to clinicians are underpinned by fears of stigmatization and the desire to normalize their illness experience by not *needing help* [20]. To support men’s psychological needs, it is essential to develop interventions that address these barriers.

### Web-Based Support

The effectiveness of cognitive behavior therapy (CBT) is well documented; it can offer an acceptable, brief intervention within mental health services for people experiencing emotional difficulties as a consequence of comorbid problem(s) [21,22]. More recently, Web-based CBT has proved as effective as CBT delivered face-to-face [23]. However, there is mixed evidence for the role of clinician guidance in Web-based interventions. Although clinician support has been considered important for beneficial outcomes, there is evidence to suggest that the level of training for those providing guidance may be of limited importance; in some cases therapist effect may be minimal, and support can equally come from nonclinicians [23,24]. Conversely, a recent review concluded that there is limited evidence to show that self-guided interventions, in any delivery mode, can reduce psychological distress after cancer, but the authors did consider that efficacy may be increased if interventions are targeted at people formally assessed as being distressed [25]. Notably, recent meta-synthesis of qualitative studies in long-term conditions established that building Web-based social ties with peers can support self-management and improve illness experiences in aspects that are hard for individuals to negotiate offline [26].

Web-based CBT for cancer patients, and prostate cancer in particular, is a less-developed area compared with other chronic physical conditions [27-31]. For men with prostate cancer, Web-based delivery of psychological support is promising, it can facilitate access and engagement by providing a faceless, perceptually private environment to ameliorate men’s fears of stigmatization; it can also prove cost-effective for health services. A systematic review has shown that psychological interventions for prostate cancer survivors can improve mental health [32], but although 10 of the 21 effective patient-focused interventions identified were based on or contained components of CBT, only 2 were Web-based interventions. Both showed an improvement in depression [33] or distress [31], but neither of the interventions was carried out among a sample of men who had been formally assessed as being distressed nor were they delivered within a clinical setting. Outcomes from these studies are more relevant to *worried* prostate cancer patients than to a clinically distressed prostate cancer population requiring a therapeutic service.

### Study Aim

In this study, we describe the development and feasibility of delivering a Web-based intervention in clinical practice for men with mild and moderate distress after treatment for prostate cancer. The program offers self-guided CBT augmented with filmed peer support and low-level chat room facilitation to encourage self-management; it aims to offer men the ability to monitor their condition and to affect the cognitive, behavioral, and emotional responses necessary to maintain an acceptable level of psychological well-being [32,34,35]. The program is intended to provide a cost-effective, brief intervention that can be offered through health services with minimal practitioner input. Reflecting recommended foci for feasibility studies [36], we assessed (1) demand through uptake and attrition, (2) acceptability by adherence and participant satisfaction, (3) potential for improvement in distress (and self-efficacy phase II) through limited efficacy testing, and (4) potential challenges for implementation in clinical practice.

## Methods

### Study Design

We conducted 2 phases of feasibility research. Phase I assessed the program prototype in a low-intensity, primary care psychological service within which it was developed. Data from that phase informed further development, and phase II tested a slightly revised version in a secondary care cancer service.

The studies were approved by the UK NHS National Research Ethics Service, phase I reference 13/SC/0065; phase II reference 15/SC/0690.

In accordance with the Medical Research Council framework for developing and evaluating complex interventions [37], we used a pre-post, within-participant comparison, mixed-methods design in both phases. In phase II, sequential qualitative interviews were conducted after the final assessments to provide complementary context to the data [38]. In phase I, the intervention ran in February and again in March 2015, with separate facilitators and participant cohorts. In phase II, the intervention ran once with a single cohort and facilitator in June 2016.

### Participants, Setting, and Recruitment

**Figure 1:**
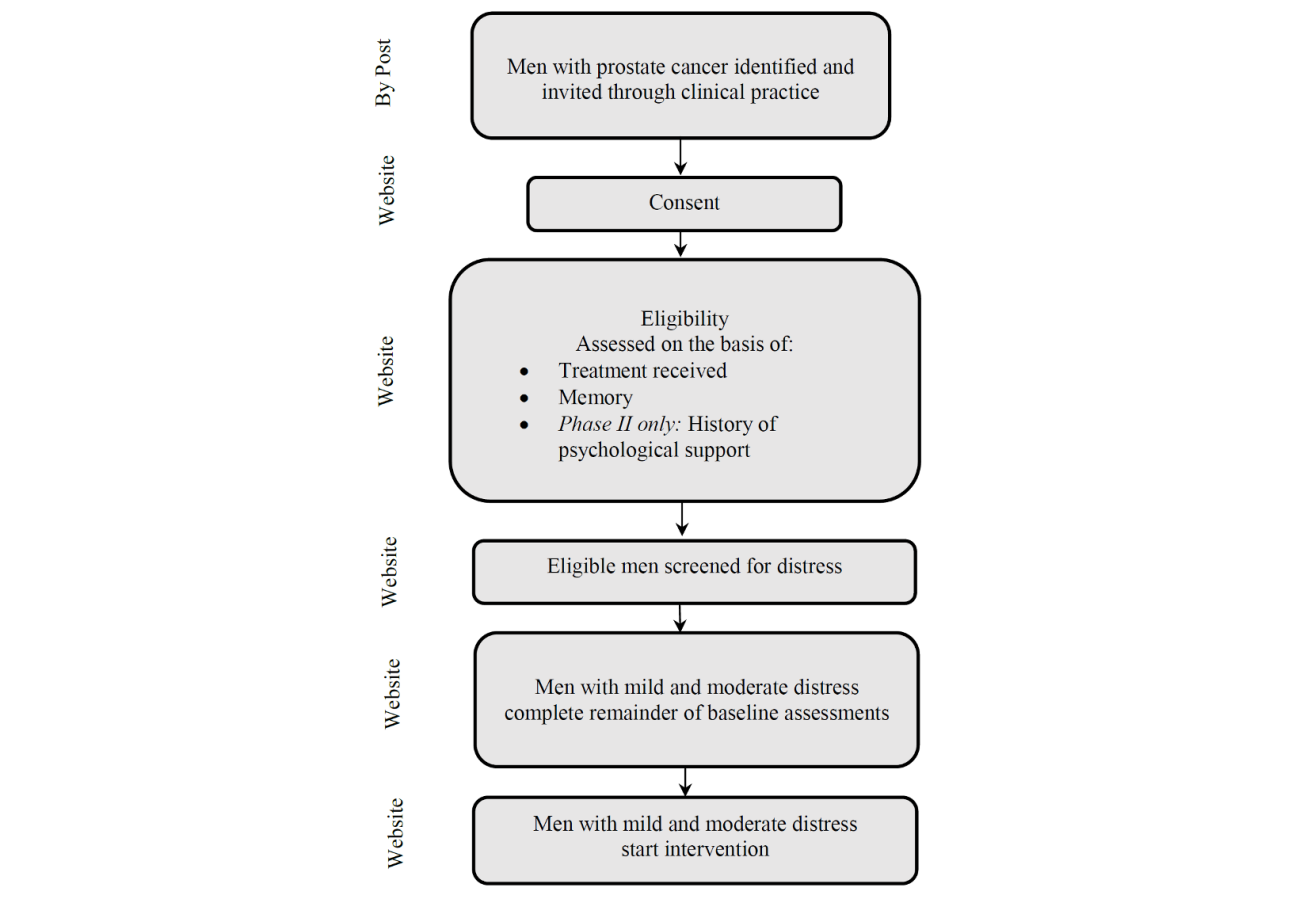
Identification, eligibility, and screening.

Identification, eligibility, and screening are outlined in Figure 1. Men diagnosed with prostate cancer, not receiving palliative care for metastatic disease, were invited. In phase I, 432 men diagnosed up to 5 years were invited by a letter from their primary care physician; in phase II, 606 men diagnosed up to 3.5 years were invited by a letter from a nurse consultant in a secondary care cancer service. The letter contained full participant information and a link to the study website where all further contact took place. Interested men visited the website and gave informed consent. Consented men were then assessed for eligibility and those eligible were screened for distress. Men experiencing mild distress and men experiencing moderate distress were asked to complete the remainder of baseline assessments and were offered the intervention. (All inclusion and exclusion criteria are summarized in Table 1.) A risk-assessment protocol was administered throughout phases I and II (see Multimedia Appendix 1).

**Table 1:** Inclusion and exclusion criteria.

Inclusion and Exclusion	Phase I	Phase II
Inclusion	Men diagnosed with prostate cancer in last 5 years	Men diagnosed with prostate cancer in last 3.5 years
	Received or receiving treatment: prostatectomy, radiotherapy, brachytherapy, hormone therapy, active surveillance, or watchful waiting	Received or receiving treatment: prostatectomy, radiotherapy, brachytherapy, hormone therapy, active surveillance, or watchful waiting
	Experiencing mild and moderate distress	Experiencing mild and moderate distress
Exclusion	Palliative metastatic disease	Palliative metastatic disease
	Referral or medication for memory loss	Referral or medication for memory loss
	Counseling or psychiatric referral since diagnosis	(Men were not excluded on the basis of counseling or psychiatric referral)
	Experiencing severe depression or suicidal thoughts	Experiencing severe depression or suicidal thoughts

### Intervention

The program concept was developed from our previous research in urinary symptom self-management after prostate cancer treatment [39]. In response to a custom-made motivational peer support film used in the randomized controlled trial of that intervention, service users requested self-guided, easily accessible support to help manage their psychological distress. This need was confirmed in a scoping review of available psychological care, literature review of the status of cancer-related psychological interventions [40], and emerging policy initiatives to provide psychological support within primary care services for people struggling with a chronic condition.

The Web-based program, Getting Down to Coping, was produced in conjunction with a low-intensity psychological service that accepted general physician and self-referrals. The service was part of England’s National Health Service, Improving Access to Psychological Therapies (IAPT) program, which offered low- or high-intensity therapy, or referral to specialist mental health services [41]. Particular features included the use of standardized and manualized evidence-based CBT and routine outcome monitoring at each clinical session. The low-intensity service typically offered brief courses for people with mild and moderate anxiety or depression, mostly by telephone, but face-to-face and computer-based support was available. Published evaluations have reported recovery rates in excess of 50%, supporting service objectives, and IAPT is now developing services to provide tailored support for people with mental health needs associated with a long-term physical condition [42,43].

The research team, in collaboration with a software engineer, senior mental health practitioners, urologist, psychosexual therapist, specialist nurse, and 3 user representatives, codeveloped the initial program prototype based on the manualized short course of CBT delivered by the service. The course was then tailored to reflect prostate cancer-related examples and concerns and supplemented with links to medical, physical, emotional, social, and financial prostate cancer information. The program website was styled with graphics and language to appeal to a male audience and to reduce connotations of mental health.

A fundamental component of the program was peer support, but providing this over the internet is complex. Active engagement with others through posting messages can mediate positive outcomes, but not all men are prepared to do this, and evidence shows that passively viewing messages is not associated with the same beneficial outcomes [31,44]. To support men, we embedded theory-driven peer support films [45], as well as a platform for interactive support via an asynchronous chat forum. A single chat forum thread ran weekly; each week the facilitator started the thread by posting a question relevant to the module topic. The program was beta-tested by service users and the films were evaluated in focus groups. Comments from users and research participants were incorporated to refine the program and films.

The program contained 4 weekly, consecutive CBT modules with an introduction at the beginning of Module 1 (see Multimedia Appendix 2). It was intended that men should spend up to an hour per week on each module, including chat room activity. Modules were available 1 each week and progress was sequential. A male narrator supported the text. Men were invited to create a profile that other men could view. All worksheets and materials were available to download.

**Figure 2:**
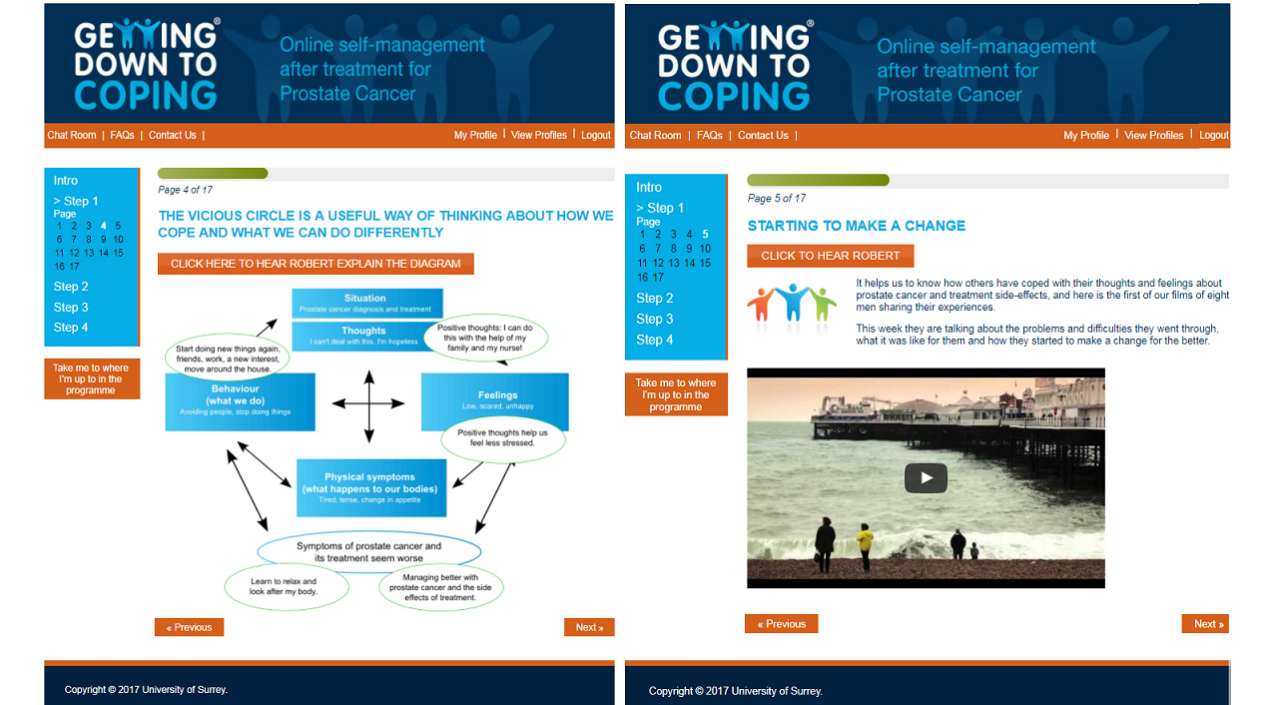
Screenshots of the Getting Down to Coping Program.

The IAPT service used the Patient Health Questionnaire-9 (PHQ-9) [46] and the General Anxiety Disorder-7 scale (GAD-7) for outcome monitoring [47]. To mirror the service’s practice, these were administered weekly within the program at the beginning of each module. Except in a situation of risk, it was intended that no formal feedback from these measures would be given to participants. In phase II, the measures were replaced with a noncompulsory weekly mood diary that invited participants to rate how they were feeling on 5 scales: down/cheerful, irritable/calm, vulnerable/in control, weary/active, and worried/relaxed; participants had the opportunity to review their previous scores. Phase II incorporated email notifications of chat room posts, chat room access from any page, easier navigation, and frequently asked questions for IT support. (screenshots in Figure 2.)

The prototype was developed with hard-coded software, which limited functionality. For phase II, the program was redeveloped within a content management system to enable integration into clinical practice and facilitate further research.

### Facilitation

There were 3 chat room facilitators, each responsible for 1 cohort of men and all were trained by a subteam of researchers, clinicians, and user representatives. Training covered Web-based facilitation, prostate cancer issues, and/or CBT theory. In phase I, 2 low-intensity psychological practitioners from the collaborating service carried out facilitation. Each practitioner allotted 2, predesignated 2-h slots per week to facilitate the chat forum for their cohort. They also visited the website intermittently during office hours to assess risk. During these slots, the practitioners did not continue with their usual clinical caseload. In phase II, a specialist cancer nurse delivered facilitation and accessed and interacted with the program on an ad hoc basis during the time it was available to participants.

### Data Collection

#### Uptake

Uptake was assessed as the proportion of men who took up the initial invitation, visited the website, and gave their consent.

#### Sample Characteristics

Demographic, disease, and treatment characteristics were collected in both phases from eligible participants before distress screening. Comorbidity was assessed separately at baseline in phase I and post intervention in phase II.

#### Screening and Outcome Assessment

Distress screening took place at baseline 2 weeks before the intervention; the remaining baseline assessments were completed 1 week before the intervention. Participants who entered the intervention were followed-up and assessed through the website in the week after intervention completion.

#### Attrition

Attrition was assessed by the number of men offered the intervention who dropped out before, during, or after the program and the proportion of core users who continued to use the program [48].

#### Adherence

Adherence is an important mediating variable for benefit in health-related Web-based interventions, yet it is a challenge to achieve and measure [49,50]. We assessed adherence using the persuasive system design (PSD) model [50,51], which proposes that the content of Web-based behavior-change programs is conveyed by a range of design features that can persuade and motivate the user without *deception, coercion or inducement* [52]. Design features can account for more than half the variance in adherence, but researchers have been slow to take account of this [50,52]. The PSD model advances 4 principles of design support through which an interactive system can persuade and enhance use: (1) support given to the primary *task* to communicate meaningful content, (2) support given to a *dialogue* between the program and the participant to help participants move toward their goal, (3) support provided through *social* features of the program to enhance participant motivation, and (4) *credibility* support that makes the system trustworthy and believable [51]. The Getting Down to Coping program contained elements of all 4 principles; in particular, quantifiable elements were located in *task support* and *social support* (Table 2). We also assessed static measures: time logged-in in phase I, time logged-in-and-active in phase II defined as follows: (1) any action within 10 min of a previous action would be considered to take place within the same session and (2) the user would be expected to look at the site for 1 min after their last action. We examined adherence among core users [48].

**Table 2:** Persuasive system design principles reflected in the Getting Down to Coping Program.

PSD^a^ principles [51]	PSD elements^a^	Getting Down to Coping Program components
Supporting the primary task	Tunneling: Using the system to guide users through a process of experience provides opportunities to persuade along the way.	Content delivered in sequential modules that can only be accessed when the system releases the next module; Opportunities to self-assess and review progress.
	Tailoring: Information provided by the system will be more persuasive if it is tailored to the potential needs, interest, personality, usage context, or other factors relevant to a user group.	Program is prostate cancer focused throughout in respect of context, examples, and suggestions; Provides targeted links to Web-based information, education, and support services.
	Self-monitoring: A system that keeps track of a user’s own performance or status supports the user in achieving goals.	In phase II, mood diary and CBT entries are available for back reference once completed.
Supporting the computer-human dialogue	Reminders: If a system reminds users of their target behavior, or that the system is ready to use, the users will more likely achieve their goals.	Emails from the system announce the imminent beginning of each module; In phase II, email notifications are sent to all chat room users when someone posts.
	Suggestions: Systems offering fitting suggestions will have greater persuasive powers.	Text and voice over provide suggestions for action.
	Similarity: People are more readily persuaded through systems that remind them of themselves in some meaningful way.	Graphics and layout are attractive and pertinent to men, films show men in similar situations, and language is inclusive and colloquial. Narrator (Robert) conveys familiarity.
	Social role: If a system adopts a social role, users will more likely use it for persuasive purposes.	Facilitator role to encourage peer support and self-management.
Supporting the credibility of the system	Trustworthiness and expertise: A system that is viewed as trustworthy and/or incorporating expertise will have increased powers of persuasion.	Badging via logos endorses clinical services and research team expertise. Narrator’s voice (Robert) is reassuring.
	Surface credibility: People make initial assessment of the system credibility based on a first-hand inspection.	Ease of log-in, secure, simplicity of instructions, and clarity of format. Up-to-date, easily accessible information and downloadable resources. In phase II, wider device compatibility, addition of frequently asked questions, access to chat room from every page, easier navigation.
	Real world: A system that highlights people or organizations behind its content or services will have more credibility.	Optional voice over throughout; possibility of contacting facilitator for private email chat.
Social support	Social learning: A person will be more motivated to perform a target behavior if they can use a system to observe others performing the behavior	Chat forum provides opportunity to interact, to discuss self-assessment and progress, and to provide or receive support.
	Social comparison: System users will have a greater motivation to perform the target behavior if they can compare their performance with the performance of others.	Participants can compare their experiences with those of their peers in the films and in the chat forum.

^a^PSD: persuasive system design. Part of table used with permission from Association for Information Systems, Atlanta, GA; 404-413-7444; All rights reserved.

#### Participant Satisfaction

Satisfaction with the program was assessed at post intervention via an author-generated questionnaire containing 4 Likert-type scales representing: (1) recruitment, (2) program, (3) chat room, and (4) films. In-depth telephone interviews were conducted by the study researcher with 10 phase II participants to understand personal experiences and contexts. Interviews were audio recorded, transcribed, and analyzed using framework analysis [53].

#### Distress

Screening for distress was measured by the General Health Questionnaire-28 (GHQ-28) [54]. Performance in cancer populations shows high reliability (rho≥.80, kappa≥.60, *r*=.8) and high validity (≥80%) [55]. Mild and moderate distress was assessed as a score ≥4.

The PHQ-9 [46] and GAD-7 [47] were administered at baseline and post intervention in both phases. In phase I, the program’s week 1 data were used as baseline.

#### Self-Efficacy

In phase II, the Self-Efficacy for Symptom Control Inventory (SESCI) was administered at baseline and post intervention to assess participant self-belief to cope and manage prostate cancer-related symptoms. The SESCI contains 3 subscales: self-efficacy for physical function, self-efficacy for coping or tolerating symptoms, and self-efficacy for symptom management. Participants indicate how confident they feel on scales for each domain from 0 (not confident) to 100 (very confident). The measure is a modified version of a self-efficacy scale used in chronic pain and lung cancer symptoms [56,57]. For prostate cancer patients, reliability has been calculated with a Cronbach alpha for the total scale of .97, and for each subscale of .94 [58].

#### Service Implementation

Time spent by the facilitators in the program was assessed by log-in data. Issues related to delivery and integration in practice were explored after the intervention: in phase I, the study researcher conducted face-to-face interviews with the 2 facilitators; in phase II, a telephone interview was conducted with the facilitator. Issues relating to delivery and integration into current practice were explored. Interviews were audio recorded, transcribed, and analyzed with framework analysis [53].

### Data Analysis

We assessed participant demographic, disease, treatment and satisfaction profiles, and uptake and adherence descriptively. We used descriptive statistics (mean, median, standard deviation [SD], interquartile range [IQR]) and box plots to examine the distribution of distress measured by the GHQ-28 and self-efficacy. The samples were not powered to detect significance in the outcome measures, nevertheless we present nonparametric data in relation to distress and self-efficacy to aid understanding of the potential effect of the program within these samples and provide data on which to base a power calculation for a larger study of efficacy. Statistical testing was performed on the 2 samples of core users, which for feasibility testing in this design provides a more useful measure of overall outcome [48].

## Results

### Uptake

**Figure 3:**
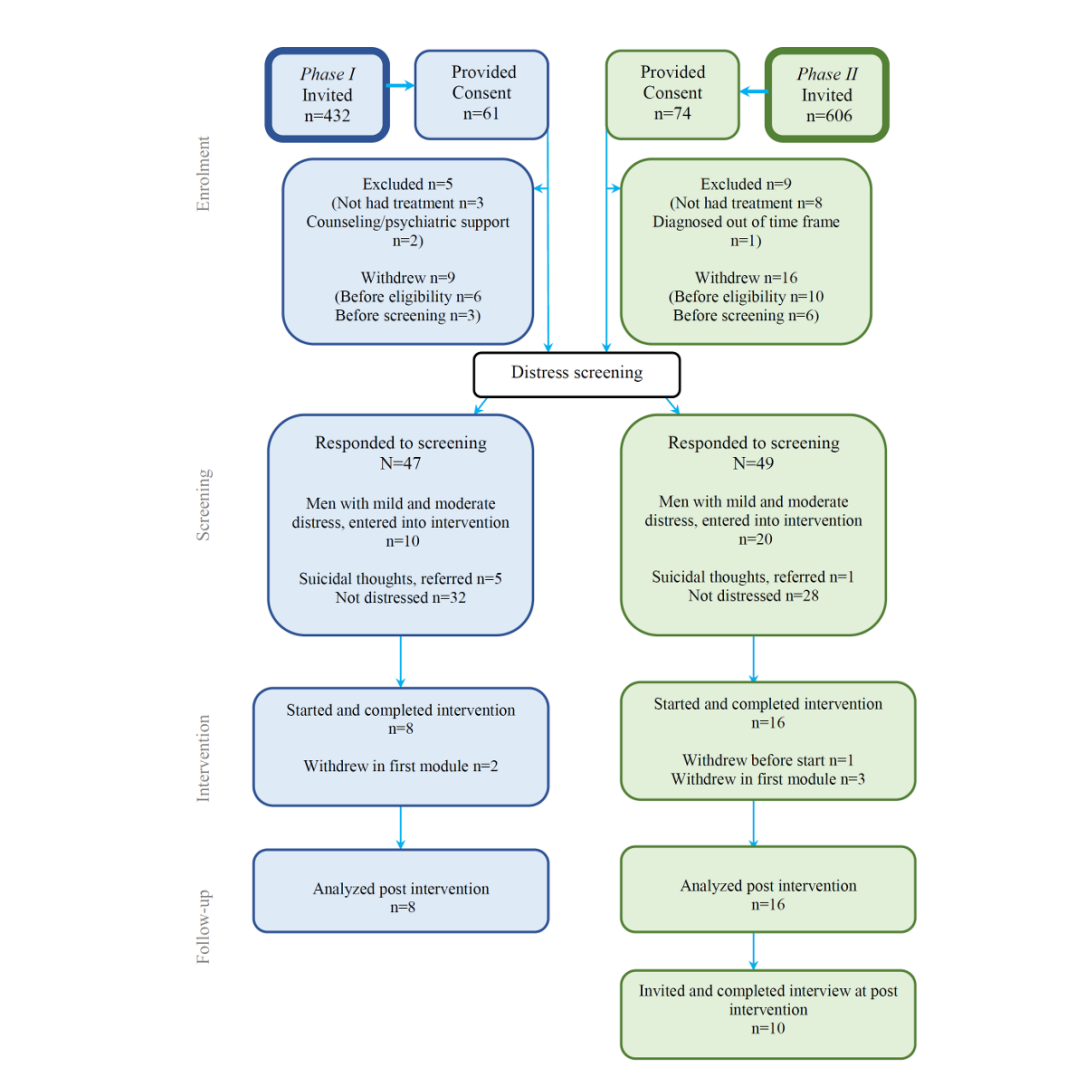
Consolidated Standards of Reporting Trials (CONSORT) diagram of participant flow.

A total of 14.1% (61/432) of invited men consented to the take part in phase I, and 12.2% (74/606) of invited men consented to take part in phase II (Figure 3).

### Participants

In phase I, 47 eligible men were screened, of whom 32% (15/47) were experiencing distress: 21% (10/47) indicated mild and moderate distress and were offered the intervention and 11% (5/47) indicated suicidal thoughts and were referred for clinical assessment.

In phase II, 49 eligible men were screened, of whom 43% (21/49) were experiencing distress, 41% (20/49) indicated mild and moderate distress and were offered the intervention, and 1 indicated a suicide risk and was referred for clinical assessment.

### Attrition

In phase I, 10 participants were offered and started the intervention, and 2 withdrew in the first module (1 felt the program was not appropriate for his needs and 1 gave no reason). A total of 80% (8/10 across 2 cohorts) remained in the program for the 4 weeks and completed all assessments (Figure 3).

In phase II, 20 participants were offered the intervention, 1 did not start (no reason given), and 3 withdrew during the first module (1 declined because he had been recently bereaved and 2 gave no reason). A total of 80% (16/20 in 1 cohort) remained in the program for the 4 weeks and completed all assessments (Figure 3).

### User Profiles

Baseline demographic, disease, and treatment characteristics for core users are reported in Table 3.

#### Demographics

In phase I, the median age was 68 years; typically, men were retired or working part-time, educated up to the age of 16 or 18 years without any higher education, and living in *least* deprived areas of the region. All were of white ethnicity, living with a partner, and reported co-existing health conditions.

In phase II, the median age was 62 years; men were typically living with a partner, retired, or working full-time; a fifth were on long-term sick leave. All were of white ethnicity, polarized between *most* and *least* deprived areas. All except one reported co-existing health conditions.

#### Disease and Treatment

The majority of men in both phases had been diagnosed under 2 years; locally confined disease at diagnosis was reported by less than half in phase I and by nearly 3 quarters in phase II. The majority in both phases recalled a PSA at diagnosis >10.

Most men in both phases were undergoing active treatment at the time of the intervention, either hormone treatment or hormone plus external beam radiotherapy.

All those who had completed treatment had done so within 1-2 years. In phase I, men had received hormone and or external beam radiotherapy; in phase II, there was a broad range of treatment experience, including prostatectomy. A minority in each phase had experience of active surveillance; 1 man in phase II was undergoing active surveillance.

### Adherence

#### Task Support

On the basis of page views, 50% (4/8) of men in phase I reached the end of all the modules. In phase II, 88% (14/16) of men reached the end of all the modules. This equates to overall module adherence rates of 63% (mean 2.5, SD 1.9) and 92%, (mean 3.7, SD 1.0) respectively.

On the basis of a possible 21 CBT entries, there was an adherence rate of 77% (mean 16.1, SD 6.2) in phase I, and 88% (mean 18.6, SD 3.9) in phase II. The mood diary in phase II was completed by 100% (16/16) of men.

#### Social Support

A total of 6 out of 8 men (75%) in phase I viewed all 4 peer support films, 1 (1/8) man watched 2 films, and 1 (1/8) man watched 3 films, equating to an adherence rate of 91% (mean 3.6 SD 0.7). Data available for the 4 weeks of phase II, indicated a total of 16, 11, 11 and 10 unique weekly views, respectively.

In phase I, 63% (5/8) of men posted in the chat room: median posts n=2 (range 1-6). In phase II, 75% (12/16) men posted: median posts n=5 (range 1-24).

#### Log-in Behavior

In phase I, median time logged in was 5 h 35 min (range 2 h 38 min to 11 h 31 min). In phase II, median time logged-in-and-active was 4 h 5 min (range 1 h 8 min to 8 h 33 min).

### Participant Satisfaction

Questionnaire responses indicated that the Web-based recruitment and consent process was understood, appropriate in language and style, swift to respond, and easy to access (Table 4). Response to the program and the films was also positive, but there were some issues raised and clarified in open-ended and qualitative responses that will inform future development. Issues were related to the following: (1) length of sessions, the last module *Getting There* was shorter than the preceding modules, which was disappointing; (2) questions about suicide (at screening and assessment) were alarming for some; and (3) the need to enhance identification with the men in the films by providing details of what treatments they had received. In phase I, the chat room was poorly endorsed: it had been difficult to locate, there had been little activity and opportunity to chat, and the facilitation was not perceived as supportive. These issues were addressed in phase II and satisfaction improved: access and ease of use was enhanced, notifications of chat room activity were provided, and facilitator interaction was increased.

**Table 3:** Core user profiles.

Core user characteristics		Phase I (N=8)	Phase II (N=16)
Age in years, mean (SD)		69 (6.1)	64 (6.9)
Age in years, median (range)		68 (61-79)	62 (55-80)
**Age groups, n (%)^a^**			
	50-59	0 (0)	4 (25)
	60-69	5 (63)	9 (56)
	70-79	3 (37)	2 (13)
	80-89	0 (0)	1 (6)
**Living status, n (%)^a^**			
	Alone	0 (0)	1 (6)
	With partner	8 (100)	15 (94)
**Working status, n (%)^a^**			
	Full-time	1 (13)	5 (31)
	Working part-time	3 (37)	0 (0)
	Long-term sick	0 (0)	3 (19)
	Retired	4 (50)	7 (44)
	Other	0 (0)	1 (6)
**Education, n (%)^a^**			
	Up to 16 years	3 (37)	12 (75)
	Up to 18 years	4 (50)	1 (6)
	Post 18 years Diploma/certificate	0 (0)	2 (13)
	Higher education	1 (13)	1 (6)
**Residential area: EIMD^b^; SMID^c^ quintiles, n (%)^a^**			
	1 Most deprived	0 (0)	3 (19)
	2	1 (13)	4 (25)
	3	2 (25)	2 (13)
	4	3 (37)	4 (25)
	5 Least deprived	2 (25)	3 (19)
**Ethnicity, n (%)^a^**			
	White	8 (100)	16 (100)
**Comorbidities, n (%)^a^**			
	0	0 (0)	1 (6)
	1	4 (50)	6 (38)
	2	3 (37)	2 (13)
	3	1 (13)	4 (25)
	4	0 (0)	3 (19)
**Time since diagnosis, n (%)^a^**			
	Under 1 year	4 (50)	7 (44)
	1-2 years	1 (13)	2 (25)
	2-3 years	0 (0)	5 (31)
	3-4 years	1 (13)	0 (0)
	5 years +	2 (25)	0 (0)
**Stage of disease at diagnosis, n (%)^a^**			
	I	3 (38)	8 (50)
	II	0 (0)	3 (19)
	III	3 (38)	4 (25)
	IV	1 (13)	1 (6)
	Missing	1 (13)	0 (0)
**PSA^d^ score, n (%)^a^**			
	<4	0 (0)	0 (0)
	4-10	1 (13)	2 (13)
	>10	6 (75)	13 (81)
	Missing	1 (13)	1 (6)
**Gleason score (biopsy), n (%)^a^**			
	6	1 (13)	2 (13)
	7	3 (43)	4 (25)
	8-9	3 (43)	4 (25)
	Missing	1	6
**Time since active treatment n (%)^a^**			
	Current treatment	5 (63)	8 (50)
	Under 1 year	0 (0)	5 (31)
	1-2 years	3 (37)	2 (13)
	Not had active treatment	0 (0)	1 (6)
**Treatment received^e^, n (%)^a^**			
	Prostatectomy	0 (0)	9 (56)
	External beam radiotherapy	4 (50)	7 (44)
	Brachytherapy	0 (0)	1 (6)
	Hormone therapy	7 (87)	10 (63)
	Active surveillance	1 (13)	6 (38)
	Watchful waiting	0 (0)	3 (19)
**Current active treatment, n (%)^a^**			
	Hormone therapy	5 (63)	8 (50)
	External beam radiotherapy	2 (25)	2 (13)

^a^Percentages rounded.

^b^EMID: English Index of Multiple Deprivation (phase I).

^c^SIMD: Scottish Index of Multiple Deprivation (phase II).

^d^PSA: prostate-specific antigen.

^e^Participants may have had, or be having, more than 1 treatment.

**Table 4:** Participant satisfaction.

Program elements: Likert Scales		Phase I (N=8), n (%)^a^			Phase II (N=16), n (%)^a^		
		Agree^b^	Disagree^c^	Neutral^d^	Agree^b^	Disagree^c^	Neutral^d^
**Recruitment pages**							
	Understood the process	8 (100)	0 (0)	0 (0)	16 (100)	0 (0)	0 (0)
	Language appropriate	8 (100)	0 (0)	0 (0)	15 (94)	0 (0)	1 (6)
	Look appropriate	8 (100)	0 (0)	0 (0)	14 (88)	0 (0)	2 (13)
	Emails swift	7 (88)	0 (0)	1 (13)	16 (100)	0 (0)	0 (0)
	Links easy to access	6 (75)	1 (13)	1 (13)	16 (100)	0 (0)	0 (0)
**The program**							
	Language appropriate	8 (100)	0 (0)	0 (0)	15 (94)	0 (0)	1 (6)
	Length of each step right	7 (88)	1 (13)	0 (0)	11 (69)	2 (13)	3 (19)
	Week per session right	7 (88)	0 (0)	1 (13)	12 (75)	1 (6)	3 (19)
	Questionnaires did not interfere^e^	7 (88)	0 (0)	1 (13)	N/A	N/A	N/A
	Mood diary was helpful^f^	N/A	N/A	N/A	7 (44)	3 (19)	6 (38)
	Worked through smoothly	6 (75)	1 (13)	1 (13)	15 (94)	0 (0)	1 (6)
	Links easy to access	6 (75)	0 (0)	2 (25)	16 (100)	0 (0)	0 (0)
	Information helpful	6 (75)	0 (0)	2 (25)	14 (88)	0 (0)	2 (13)
	Understood diagrams	5 (63)	0 (0)	3 (38)	16 (100)	0 (0)	0 (0)
	Worksheets useful	5 (63)	0 (0)	3 (38)	7 (44)	1 (6)	8 (50)
	Robert’s voice helped me	4 (50)	0 (0)	4 (50)	10 (63)	1 (6)	5 (31)
**Chat forum**							
	Easy to locate	3 (38)	2 (25)	3 (38)	13 (81)	0 (0)	3 (19)
	Facilitator was supportive	2 (25)	1 (13)	5 (63)	9 (56)	0 (0)	7 (44)
	Opportunity for private chat was reassuring^g^	1 (13)	1 (13)	6 (75)	7 (44)	0 (0)	9 (56)
	Learned a lot from other men	0 (0)	3 (38)	5 (63)	10 (63)	1 (6)	5 (31)
**Films**							
	Range of experiences and stories	7 (88)	0 (0)	1 (13)	15 (94)	0 (0)	1 (6)
	Made me feel not alone	7 (88)	0 (0)	1 (13)	10 (63)	0 (0)	6 (38)
	Program benefited from films	6 (75)	0 (0)	2 (25)	12 (75)	0 (0)	4 (25)
	Men were representative	5 (63)	0 (0)	3 (38)	14 (88)	1 (6)	1 (6)
	Could relate to men	5 (63)	2 (25)	1 (13)	11 (69)	2 (13)	3 (19)
	Reflected learning in modules	4 (50)	0 (0)	4 (50)	10 (63)	0 (0)	6 (38)
	Gave me confidence^h^	4 (50)	0 (0)	4 (50)	9 (56)	0 (0)	7 (44)

^a^Percentages rounded.

^b^Agree + agree strongly.

^c^Disagree + disagree strongly.

^d^Neither agree nor disagree.

^e^Phase I only.

^f^Phase II only.

^g^Full item *Opportunity to have private chat with facilitator was reassuring*.

^h^Full item *Gave me confidence to make a difference to how I feel*.

**Figure 4:**
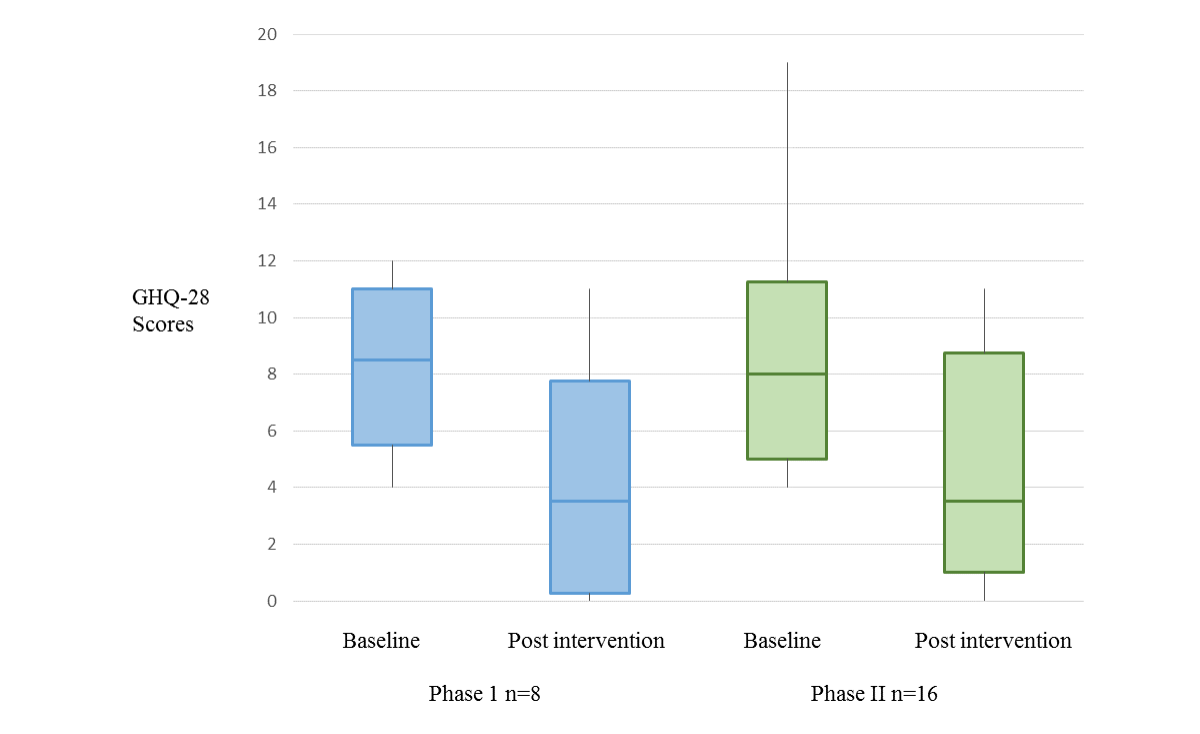
Change in distress. General Health Questionnaire-28 (GHQ-28).

Phase II interviews helped clarify the questionnaire responses. (Participant profiles: Multimedia Appendix 3; verbatims: Multimedia Appendix 4). Acceptability of the program was high, even among those with low IT skills, and there was a readiness to improve skills. Men were comfortable using tablets, mobile phones, laptops, and desktop computers: they mainly found it effortless and flexible in comparison with other forms of support, even those who were finding concentration, or the availability of free time, a challenge. Some expectations were not met: disappointment with the length of modules noted in the assessment was attributed to less interaction time and *things to do* in the final module.

Men readily identified that the program targeted issues they found difficult to talk about. Learning about the link between the effects of treatment and mood and behavior was a fresh perspective, and they felt that the skills developed to manage now would be useful should things change in the future. Men referred more consistently, however, to other aspects of the program. The weekly provision of information via a range of links related to that week’s learning was used enthusiastically because it provided access to immediate information that meant men could control when and how much they consumed. They identified that having the links was an improvement on their usual Internet use because they were direct and avoided lengthy searching and inappropriate or potentially scary information. The films provided discreet stories, preventing the unpredictable, which helped men feel connected and in control, particularly those with social and information needs.

For those who engaged in the chat room, it was a safe environment where they could be honest with each other without the inhibitions they often experienced with clinicians. It was also a source of quick answers to spontaneous questions, which for some was very appealing; for others, it could be daunting if something was revealed that was incorrect or alarming. Despite the improved chat room satisfaction scores in phase II, some felt that the facilitator could have made more attempts to encourage men to open up and interact.

### Distress

The samples were not powered to detect a significant change. Notwithstanding, we carried out nonparametric testing to determine potential for change in distress between baseline and post intervention; the samples performed similarly (Figure 4). A Wilcoxon signed-rank test indicated improvement in distress at the end of the intervention in both phases (phase I *z*=−2.213, *P*=.03, *r*=−.55; phase II z=−3.342, *P*=.001, *r*=−.59). In phase II, we also calculated change in domain scores. From baseline to post intervention, there was a positive change in somatic domain symptoms (*z*=−2.588, *P*=.01, *r*=−.458) and anxiety domain symptoms (*z*=−3.466, *P*=.001, *r*=−.613). Scores for social dysfunction domain symptoms were *z*=−1.531, *P*=.13, *r*=−.27 and for severe depression domain symptoms scores were *z*=−1.283, *P*=.20, *r*=−.23 (see Multimedia Appendix 5).

#### Clinical Caseness

A total of 17% (4/24) of participants overall registered scores on the PHQ-9 and GAD-7 that were above the clinical threshold for depression and anxiety and defined them as cases requiring clinical intervention in accordance with IAPT protocols. Of these 4, 3 scored over the threshold for depression on the PHQ-9, and 3 scored over the threshold for anxiety on the GAD-7 (see Multimedia Appendix 6).

### Self-Efficacy Phase II

At baseline, men were most confident in performing daily activities (mean 69, median 80.0, SD 22.4, range 18-72), less-confident coping/tolerating symptoms (mean 52, median 53.5, SD 17.2, range 26-80), and least confident managing their symptoms (mean 31.6, median 31.50, SD 9.3, range 12-49; see Multimedia Appendix 7). A Wilcoxon signed-rank test performed on baseline and post scores showed an improvement in coping (z=−2.329, *P*=.02, *r*=−.412). Change was not indicated in managing symptoms (*P*=.11) or in performing daily activities (*P*=.08).

### Service Implementation

#### Facilitator Time

Phase I facilitator clinical time was ring-fenced for 2, 2-h sessions per week, that is, 16 h over a 4-week program. To risk assess and monitor the program at other times, 1 facilitator spent 6 h 56 min in the program and the second spent 8 h 29 min, giving a total of 22 h 56 min and 24 h 29 min, respectively, per program. In phase II, the facilitator spent 15 h 45 min in total across 1 program.

#### Facilitator Feedback—Delivery

The psychological practitioners were reassured the program was consonant with their standard CBT practice. They emphasized that the lay approach did not overwhelm participants and encouraged active log-in and participant commitment. They indicated their clients generally found it difficult to differentiate the effects of physical and psychological symptoms on mood, and often there was little change on the service’s standard outcome measures for clients with a physical long-term condition. In the cancer service, the opportunity to offer evidenced-based support for distress was welcomed as a practical and timely benefit for patients; this need was considered poorly covered in the nurse-patient interaction through a lack of competences and provision (Verbatims: Multimedia Appendix 8).

#### Facilitator Feedback—Implementation

The psychological practitioners found the self-management role difficult to integrate into their skill set, and they lamented the move away from their therapeutic expertise. They were supportive of being allocated time slots for facilitation as it was necessary for case management, but there had been little need for them to respond during these times as men’s log-in preferences did not correspond to their availability. They felt this impeded the flow of conversation and highlighted the benefits of providing a rolling chat room rather than starting afresh each week. Integration into the nurse’s current practice was challenging; the accepted nurse role of *fixer* was replaced in this context by an enablement approach which was unfamiliar and was considered to require a shift in practice values calling for bespoke training. Notwithstanding, the ad hoc facilitation had enabled a flexible response, and the role had been easily assimilated into the nurse’s workload. In both services, the facilitator role was considered not to require the higher level skills associated with psychological practitioner and specialist nurse roles (Verbatims: Multimedia Appendix 8).

## Discussion

### Principal Findings

This is the first study, to our knowledge, to assess the feasibility of delivering Web-based CBT support in clinical settings among men screened with distress after treatment for prostate cancer. The Getting Down to Coping Program was embraced and acceptable to its target users. It can be delivered in a clinical service and has the potential to provide a therapeutic psychological service remotely.

#### Demand

Demand for the program was evident. Among men who were eligible and screened with mild and moderate distress, 29 of the 30 (96%) started the program. This exceeds reported rates in comparable cancer and prostate cancer Web-based studies for distress where enrolment after screening, which excluded distress, was between 31% and 41% [33,59]. Our retention rate of 80% meets the 70% criteria for feasible retention set by Yanez et al [33], and our 20% attrition rate is also at the lower end of rates found in randomized trials of Internet-based interventions for anxiety and depression, which range from 1% to 50% [60].

Initial uptake to our invitation of 12-14% among an unscreened sample, however, was low. Comparison with other Web-based, distress-related prostate cancer studies is problematic as they do not report the base numbers from which their screened samples were drawn [31,33]. Yet this level of uptake is not completely surprising. Across the spectrum of cancer, the profile of older age and male gender has been associated with lower uptake of Web-based psychological support [59]. Furthermore, mental health–related stigma can deter help-seeking behavior, particularly in men, and in a clinic environment, it has been reported that only 20% of unscreened cancer patients accept psychological help [61,62]. The remote recruitment process we used would also make it easier for reluctant men to avoid support [63].

Uptake may be enhanced if the nature and benefits of psychological support are conveyed so that accepting it is perceived as less *risky*. Our recruitment materials were intended to reduce perceptions of mental health and stigmatizing signals, but the research focus and length could have been burdensome for some. Information that is focused on the health problem rather than the trial process, and that is also brief and relatively simple, has been associated with enhanced recruitment rates [64-66]. To involve men, one approach may be to reflect the way they think and feel about receiving help. In the phase II interviews, men talked about how they were empowered rather than how they were supported by the program. This reflects work by Clover and colleagues who found in a survey among oncology outpatients the most common barrier to accepting psychological support was a preference for self-help [67]. The opportunity for men to increase control of their daily lives by self-help is a fundamental focus of the Getting Down to Coping Program and could be incorporated more explicitly into study communications. Framing the intervention in a self-management paradigm rather than a psychological one could help normalize men’s engagement. Further ways to enhance uptake would be to provide the main component of participant information over the internet with interactive elements so that men can choose what and how much to read. Clinician endorsement of the program as a self-help opportunity is another component that could encourage more men to take part [66,68]. The way in which we communicate psychological Web-based provision may be a crucial element in encouraging uptake of support and is an area for further examination [69].

#### Acceptance

Usage of the program and satisfaction of participants indicated that it is appropriate and acceptable to its core users. The adherence rates we achieved, from 63% to 100% across task and social support elements, illustrated that commitment was relatively high. In review of Web-based mental health programs, and in a trial among men with prostate cancer, rates of between 50% and 70% have been reported [31,60]. Men’s satisfaction and involvement were borne out by their willingness to improve their IT skills to get the most out of participation. The program offered experiences that were consonant with masculine ideals, for instance, being connected, acquiring tools and information, and a focus on *the self*, which increased feelings of physical, social, and emotional control. This is consistent with the notion that support programs need to reflect masculine ideals to involve men and optimize benefit [70,71].

We found a larger proportion of men with mild and moderate distress in the second phase of our research (21% and 41%, respectively), and also higher adherence rates in this second phase. There was some previous experience of psychological support in phase II, and adherence would have been enhanced by improvements to the program between phases. However, the higher distress levels and adherence could also be evidence of a greater level of commitment to the program in men of lower socio-economic status who characterized the phase II sample. These men experience poorer access to support and higher-than-average psychological need, indicating that regional differences will be an important consideration in further research and clinical implementation [9,72,73].

#### Limited Efficacy Testing

We found improvement in distress with a medium-large effect size in each phase. Particular improvements were in the somatic and anxiety domains of the GHQ-28. The nature and definition of distress is complex in a cancer population [74,75], and there have been calls for a more realistic framework to identify cancer-related distress [76]. The change in somatic symptoms confirmed that this can be a factor in the etiology of distress in a prostate population and is important to include when assessing distress [77]. The finding that only 4 of our participants would have been offered standard psychological support on the basis of assessment with the PHQ-9 and GAD-7, which do not include somatic symptoms, further suggests that more tailored tools are required for this population.

We also found an improvement in confidence to cope with prostate cancer symptoms but not for confidence to perform daily activities or to manage symptoms. This can be expected; performing daily activities was at a high level at baseline, leaving little room for improvement, whereas physical symptoms related to the longer-term effects of prostate cancer treatment can often be intractable [78]. Ongoing rehabilitation has to be focused on building resilience and fostering understanding and coping with symptoms. The program can offer this focus for men.

#### Implementation

Implementing the Getting Down to Coping Program has potential within both primary and secondary care settings. The intervention is a self-guided program, but some facilitation is optimal for risk monitoring and would be expected by a psychological service. However, the facilitation required calls for neither advanced psychotherapeutic skills nor high-level nursing skills, only the ability to perform the core skills necessary to motivate self-management and to monitor risk. In both settings, our facilitators were not practiced in communicating within Web-based support programs, and had no previous experience of supporting self-management. Our training contained elements of both, but all the facilitators still had difficulty performing the role; greater emphasis in training on facilitating self-management via Web-based interaction is required. Notwithstanding this, facilitation may be delivered in either setting by a lower band, health support role.

Although we were not able to assess cost-effectiveness in these studies, the time spent facilitating in each service suggests that the flexible model of intervention interaction may have the greatest potential: it did not disrupt the facilitators’ standard caseload and, for the sample we had (n=16), amounted to 1 h per participant per 4-week program. This would be inversely related to the number of men in each program.

### Limitations and Strengths

There are limitations to these studies. The sample sizes were small and were not powered to detect change, and participants were not randomized. Generalization of our findings must therefore be cautious. Where we found change we do not know what variables are responsible; natural recovery could play a role and so could extraneous events. Nevertheless, with feasibility testing in 2 clinical settings, we have developed our knowledge of both the intervention and research required to move to the next trial stage. The consistency across our 2 samples on a number of measures, and the effects found, indicate that larger scale, evaluative research is justifiable. Our further research will include a longer follow-up period to provide an indication of maintenance of change, as well as full cost-effectiveness analysis. Furthermore, we will analyze covariance in respect of facilitator and group effects.

A strength of our studies was that we incorporated the theoretical model of PSD [52], which can provide an objective understanding of adherence. We were able to measure *social* and *task* design elements, which we posited were the most important features in our intervention for effecting behavior change. Measuring *intended* use is reflective of assessing compliance in face-to-face therapy and has been proposed as the most realistic reference standard for adherence in Web-based interventions [50,60]. Analysis on this basis offers more robust comparability within and across studies than static measures of exposure, such as log-in data, which are inherently subject to system and participant ambiguity. For instance, *log-in time* in phase I gave little indication of what interaction took place. In phase II, we measured *time logged-in-and-active*, which gave a clearer indication for adherence purposes, but we still had to make assumptions, that is, how long a log-in was deemed to contain *active* time. Such assumptions are often not reported in studies. More extensive application of the theoretical underpinnings of how design and system components can influence behavior change is called for [79].

### Conclusions and Recommendations for Future Research

To integrate psychological services for cancer patients in the existing care pathways, interventions that fit with health provider parameters of care provision are required [75]. Without research sampling based on defined need, or analysis of implementation processes, many intervention studies do not provide sufficient evidence of viability or efficacy [75]. We have taken the first steps to address this with a Web-based intervention, by assessing feasibility among a population that requires clinical support and by providing that support within a clinical practice context.

The program has the potential to fit within a stepped model of care by providing psychological support for men who are mild or moderately distressed and who fall within the clinical parameters for low-intensity support. Addressing these men’s needs will prevent escalation of symptoms and the need for higher-intensity therapy. Potentially, this would lead to service cost savings in terms of reduced physical and mental health service use.

In a stepped model of therapeutic care, the program requires low-level facilitation for monitoring risk, which raises cost implications versus a completely automated system. However, within the clinical services we researched, risk surveillance was a mandatory requirement, and the true cost comparison would be versus a therapist-led, face-to-face, or telephone approach. The numbers of men who can be supported with the Web-based program at any one time is subject to economies of scale, and cost advantage can increase exponentially with the volume of patients taking part. Furthermore, facilitation need not be carried out by advanced practitioners, which contributes to delivery cost advantage. Assigning staff to the facilitator role who possess appropriate competencies, as well as ensuring their involvement with and commitment to the innovation, will be crucial to its success [80].

Clinical effectiveness, and cost-effectiveness in terms of clinical delivery and health service utilization, need to be tested in an evaluative research design. Future research should be underpinned by exploratory enquiry to establish the most relevant and engaging ways to communicate study and intervention characteristics for this prostate cancer population. In future, we hope to develop the Web-based program for men with other cancers.

## Appendix

Multimedia Appendix 1 Getting Down to Coping risk assessment.

Multimedia Appendix 2 Getting Down to Coping program modules.

Multimedia Appendix 3 Phase II interview participant profiles.

Multimedia Appendix 4 Phase II participant verbatims.

Multimedia Appendix 5 Distress total change scores in phase I and total and domain change scores in phase II.

Multimedia Appendix 6 Patient Health Questionnaire-9 (PHQ-9) and General Anxiety Disorder Scale-7 (GAD-7): number of participants scoring in each diagnostic category in phase I and phase II.

Multimedia Appendix 7 Self-Efficacy change scores in phase II.

Multimedia Appendix 8 Phase I and phase II facilitator verbatims.

## Abbreviations

CBT: cognitive behavior therapy
EMID: English Index of Multiple Deprivation (phase I)
GHQ-28: General Health Questionnaire 28
IAPT: Improving Access to Psychological Therapies
PSA: prostate-specific antigen
PSD: persuasive system design
PHQ-9: Patient Health Questionnaire 9
GAD-7: General Anxiety Disorder Scale 7
SESCI: Self-Efficacy for Symptom Control Inventory
SIMD: Scottish Index of Multiple Deprivation (phase II)
