# Supplementary material for: A Web-Based Intervention to Reduce Distress After Prostate Cancer Treatment: Development and Feasibility of the Getting Down to Coping Program in Two Different Clinical Settings
Source: JMIR Cancer. 2018 Apr 30;4(1):e8. doi: 10.2196/cancer.8918 (PMC5952123; doi:10.2196/cancer.8918)
Supplement: Multimedia Appendix 1 [file cancer_v4i1e8_app1.pdf]

**Multimedia Appendix 1.**

Getting Down to Coping® risk assessment.

At screening for distress, men were excluded and referred to their general practitioner if they registered severe depression and/or suicidal intent on the GHQ-28. Further risk assessment protocol determined that men who registered severe depression and/or suicidal intent at post-intervention assessment would be referred to their GP but, with their consent, their data would be included in the study.

Monitoring of risk during the intervention was carried out by the facilitator through checking responses to the PHQ-9 and GAD-7 (Phase I), or mood diary (Phase II) as well as to programme entries and/or chat room posts. If risk was identified, the facilitator would discuss the event with their supervisor and if considered appropriate would intervene by asking the participant for a separate telephone conversation and, if necessary, referring on to appropriate services. These participants would be withdrawn from the intervention but, with their consent, their data would be included in the study.
