# Supplementary material for: A Web-Based Intervention to Reduce Distress After Prostate Cancer Treatment: Development and Feasibility of the Getting Down to Coping Program in Two Different Clinical Settings
Source: JMIR Cancer. 2018 Apr 30;4(1):e8. doi: 10.2196/cancer.8918 (PMC5952123; doi:10.2196/cancer.8918)
Supplement: Multimedia Appendix 2 [file cancer_v4i1e8_app2.pdf]

Multimedia Appendix 2.

Getting Down to Coping® Programme modules.

Module	Content	Tool, entries and assessments
**Week 1** *Introduction*	Aim and outline of programme; introduction of optional, male narrator; invitation to set up and view personal profiles; looking at how you are feeling at the moment and introduction to *low mood* and *worry*.	
**Week 1** *Getting Started*	Introduction to the Vicious Circle and the interaction between thoughts, feelings, behaviours and the prostate cancer situation.<br>Peer support film: *Getting Started* showing eight men talking about how they have been affected by prostate cancer and how they have coped and made a difference.<br>Chat Forum: Topic for the week: What's difficult for you?<br>Problem identification and goal setting.<br>Targeted links to information and guidance about Prostate Cancer.	Assessment: PHQ9- GAD-7 (Phase I) or Mood Diary (Phase II).<br>Self-assessment: Thoughts, Feelings, Behaviour, Physical Symptoms, Prostate Cancer Symptoms.<br>Record/self-assess: My problem summary.<br>SMART Goals Worksheet.<br>Self-assessment: Main Goal and smaller goals.
**Week 2** *Getting Control*	Understanding the Behaviour element of the Vicious Circle and its relationship with prostate cancer symptoms<br>Understanding and giving guidance on : Withdrawal, Avoidance and Boom and Bust.<br>Peer support film: *Getting Control* showing men talking about what they did to make a difference and get back in control of their lives.<br>Chat Forum: Topic for the week: What have you been withdrawing, avoiding or pushing yourself too hard on?<br>Understanding and Managing Physical Symptoms of Prostate Cancer Treatment side-effects.<br>Targeted links to help make a difference.<br>Relaxation.<br>Breathing Exercises.	Assessment: PHQ9- GAD-7 (Phase I) or Mood Diary (Phase II).<br>Self-assessment: Your thoughts.<br>Withdrawing worksheet.<br>Withdrawing diary planner.<br>Avoiding worksheet.<br>Avoiding diary planner.<br>Pace yourself diary planner and example.<br>Breathing exercise sheet and MP3 file.<br>Deep muscle relaxation exercise sheet and MP3 file.<br>Record/self-assess: This week I will try.
**Week 3** *Getting Positive*	Understanding the Thinking element of the Vicious Circle and its relationship with prostate cancer symptoms.<br>Techniques to manage worry and negative thoughts.<br>Peer support film: *Getting Positive* showing men talking about their worries and how they turned their thinking around, plus the importance of relationships and getting it right.<br>Understanding and guidance on relationships and communication.<br>Targeted links to get talking.<br>Chat Forum: Topic for the week: Relationships. How are you and the people who are important to you coping with your diagnosis and symptoms?	Assessment: PHQ9- GAD-7 (Phase I) or Mood Diary (Phase II).<br>Worry tree.<br>Problem solving worksheet.<br>Thought record sheet and example.<br>Self-assessment: Feelings, Thoughts, Behaviours.<br>Self-assessment: My small steps to opening up and sharing.<br>Self-assessment: This week I will try…
**Week 4** *Getting There*	Reviewing the programme.<br>Peer support film: *Getting There* showing men talking about how they have moved on to a fulfilled life.<br>Reviewing learning.<br>Chat Forum: Topic for the week: How have you found the programme? How are you coping now? Have you developed ways of managing that are helpful? How are you feeling about coping over the next few months?<br>Making a Wellness Plan.<br>Top Twelve Tips for Getting Down to Coping.<br>Targeted links to get the right help and support.	Assessment: PHQ9- GAD-7 (Phase I) or Mood Diary (Phase II).<br>Reviewing experience.<br>Self-assessment: Have you moved any further towards your goals?<br>What have you learnt from the programme?<br>Where would you like to be in three months' time?<br>Are there any techniques or information from the programme that would help you to reach these aims?<br>Wellness plan.<br>Download Top Tips.
