# Supplementary material for: A Web-Based Intervention to Reduce Distress After Prostate Cancer Treatment: Development and Feasibility of the Getting Down to Coping Program in Two Different Clinical Settings
Source: JMIR Cancer. 2018 Apr 30;4(1):e8. doi: 10.2196/cancer.8918 (PMC5952123; doi:10.2196/cancer.8918)
Supplement: Multimedia Appendix 3 [file cancer_v4i1e8_app3.pdf]

Multimedia Appendix 3.

Phase II interview participant profiles.

Participant	Age (yrs)	Disease Stage	Months since diagnosis	Treatment [1]	Active Treatment Status during Intervention
1	63	IV	19	RP and EBRT	Completed
2	64	III	17	RP and HT	Receiving
3	80	II	6	EBRT and HT	Receiving
4	76	I	29	AS	None
5	60	III	14	EBRT and HT	Receiving
6	62	III	11	RP, HT, EBRT	Receiving
7	64	I	6	RP	Completed
8	56	III	8	RP	Completed
9	76	I	5	HT	Receiving
10	59	I	30	BT	Completed

[1] RP: Prostatectomy; EBRT: External Beam Radiotherapy; BT: Brachytherapy; HT: Hormone Therapy; AS: Active Surveillance
