# Supplementary material for: A Web-Based Intervention to Reduce Distress After Prostate Cancer Treatment: Development and Feasibility of the Getting Down to Coping Program in Two Different Clinical Settings
Source: JMIR Cancer. 2018 Apr 30;4(1):e8. doi: 10.2196/cancer.8918 (PMC5952123; doi:10.2196/cancer.8918)
Supplement: Multimedia Appendix 4 [file cancer_v4i1e8_app4.pdf]

Multimedia Appendix 4.

Phase II participant verbatims.

About the Films

"I thought there was just enough information in them, they were not too long and they gave you an insight into other people. […] it's not face-to-face but you can see the guy, you can see the other man, you can see him working in the field, you can see him doing his cooking and the other boy feeding his chickens and all that. You can relate, you know you can relate to that person […] you can say that person's well, he's alive, he's a human being, he's a person, you know. If the videos weren't there you would not know who you're talking to sort of thing […]. And that was one of the actual things I looked forward to, to doing the programme, was the actual video." (P1)

" […] it made me feel that if they can, if they could cope with it and move on then I'm sure that I could of done the same thing." (P7)

"I think the videos were good for generating discussion and generating questions that people want to ask." (P5)

About the Chat Room

"[…] you were able to talk to other guys that are on the same wavelength as you […]. We were talking the way men talk, not the way that you would have to watch your Ps and Qs to a doctor or a female nurse or whatever […]. We sort of just gelled, the crowd of us that was kinda talkng together. I felt as if we could meet each other in the street and have a good chat […]. Somebody would ask a daft thing that might sound to somebody but that person did not know and they got the answer to it or they found out how somebody else was dealing with it, it was like a light bulb moment. When you take the medical terminology out of fit and you use your own sort of terminology it can help a lot to break the barrier down to starting to talk about it […]." (P6)

"[If you have] a problem or a doubt, it's only a couple of lines of text and you can sometimes get the answer that you're looking for […]." (P6)

"I expected the facilitator to be reading the posts and if something came up that maybe needed a more expert view or some advice that they might do that. But then they may have done it privately, I don't know." (P5)

"I mentioned the ED [erectile dysfunction] side [of things] […] cause I'm 56 years old, you know what I mean […] but things like that, there was nobody else coming back with that kind of experience, it just kinda died a death, I mean [I felt that the facilitator] could of taken that on-board, I wouldn't have minded and said […] 'what do you guys think about ED?' and start a thread of conversation ." (P8)

About Adherence

"I actually looked forward to doing it […] aye, every time I had to sit down and do it I was actually pleased, I thoroughly enjoyed that […] and she [wife] was sitting in the conservatory having her tea and whatever, her coffee, and reading her paper or whatever, and I'd be sitting with the iPad [reading the programme]. Yeah, the wife bought me an iPad, I'm not that eh computer literate, I'm no [good] with the likes of emails and all that, my wife does all that, I couldn't do that […]." (P1)

"I had to make time for it, consciously make time for it. Ehm, I am very busy in my job, not only when I am at work but at home as well, but I just had to make time to do it. I didn't find it a chore, I enjoyed it while I was doing it, I found it very interesting. I did the main work once a week and any time I got an email prompt, if I was at work I just checked it on my phone, if I was at home I'd probably go back in and have a look." (P5)

"I am not sure that I would go to somebody face-to-face. I tend to stick to my own resources […] it was something that I wouldn't normally do unless I was given that opportunity, which I was, so yeah, I liked it." (P8)

About Amount To Do

"I honestly thought it would be something different. I don't know what I was expecting really, […] I thought there would have been more input, i.e. more - how can I say - more things for us to do. I felt some of it […] was just a kinda tick the box, if that makes sense[…] I felt as though maybe about week four there was nothing to do, just to read and tick […]" (P8)

About Being Able to Open Up

"[…] the online programme allows you to say things or answer questions in a more honest manner, sometimes when you're face-to-face and there's a question asked you might be a little bit apprehensive about telling the truth […] . If it's involving sexuality, you know, then you would maybe feel a little bit apprehensive, but [with the programme] there was no inhibitions about answering these questions as clearly and correctly as you could at that particular time, and [to talk about] how it affected you […]. It allowed me to, to bring out, to express […] my feelings about how I was going to cope, how would my wife cope [...]." (P7)

"[…] and talking about their […] urinary problems and their bladder problems, the dysfunction and all that, it's a bit easier, I think it's easier[…] online […] you're not actually seeing the person face-to-face and you can, you can actually open up more […] (P1)

“I actually did feel a lot better for that, I did feel I wasn’t the only person in the world that had prostate cancer, it wasn’t just happening to me it was happening to other people.” (P1)
**About Accessibility and Targeting**
“It puts things into perspective - life isn’t over, there are lots of opportunities still available to you, but you have to make the decision yourself to pursue those opportunities. […] it was accessible to more or less everyone, even if you had people with language difficulties the fact that it was read out to you gave it a huge advantage I would say. […] It was well targeted at people with prostate cancer and well targeted at what people wanted to know about the disease and also about how people could lift their mood.” (P5)
**About the Information**
“[…] I had already been on the various other websites for prostate cancer, I’d used them quite a bit […] so I knew where to find information. In terms of the links provided in the programme, … [on] the internet in general, there’s tonnes and tonnes of information that you have to plough through to get to what you are looking for which has good points and bad points.   The good point is that you are learning about other aspects of it, the bad point is that you are learning about other aspects that maybe you would rather that you didn’t learn about.   So what I found very useful with [the programme] was the fact that you could go to the particular area that interested you.” (P10)
