# Supplementary material for: A Web-Based Intervention to Reduce Distress After Prostate Cancer Treatment: Development and Feasibility of the Getting Down to Coping Program in Two Different Clinical Settings
Source: JMIR Cancer. 2018 Apr 30;4(1):e8. doi: 10.2196/cancer.8918 (PMC5952123; doi:10.2196/cancer.8918)
Supplement: Multimedia Appendix 5 [file cancer_v4i1e8_app5.pdf]

Multimedia Appendix 5.

Distress total change scores in Phase I and total and domain change scores in Phase II.

GHQ-28 Scores[1]	Median	Mean	SD	Min	Max	IQR	Wilcoxon Signed-Rank Test z	p	Effect Size Pearson's r
**Total Scores**									
**Phase I** N =8									
Baseline	8	8	3.217	4	12	4.5/11			
Post	3.5	4.25	4.132	0	11	0.25/7.75	-2.213	.03	-.55
**Phase II** N=16									
Baseline	8	8.69	4.729	4	19	5/11.25			
Post	3.5	4.25	3.992	0	11	1/8.75	-3.342	.001	-.59
**Domain Scores**									
**Phase II**									
**Somatic**									
Baseline	2.25	2.83	1.873	0	7	1.01/4.02			
Post	0.51	1.45	1.753	0	5	0.01/3.01	-2.588	.01	-.458
**Anxiety**									
Baseline	2.02	2.83	2.171	0	7	1.01/4.77			
Post	0.01	0.95	1.392	0	4	0.01/1.76	-3.466	.001	-.613
**Social Dysfunction**									
Baseline	2.02	2.83	2.139	0	7	1.01/4.77			
Post	1.01	1.77	2.052	0	6	0.01/3.02	-1.531	.13	-.27
**Severe Depression**									
Baseline	0.01	0.26	0.443	0	1	0.01/0.75			
Post	0.01	0.13	0.498	0	2	0.01/0.01	-1.283	.20	-.23

[1] General Health Questionnaire-28 (GHQ-28) Total Score = 28. Four Domains each scored 1-7
