# Supplementary material for: A Web-Based Intervention to Reduce Distress After Prostate Cancer Treatment: Development and Feasibility of the Getting Down to Coping Program in Two Different Clinical Settings
Source: JMIR Cancer. 2018 Apr 30;4(1):e8. doi: 10.2196/cancer.8918 (PMC5952123; doi:10.2196/cancer.8918)
Supplement: Multimedia Appendix 6 [file cancer_v4i1e8_app6.pdf]

Multimedia Appendix 6.

Patient Health Questionnaire-9 (PQH-9) and General Anxiety Disorder Scale-7 (GAD-7): number of participants scoring in each diagnostic category in Phase I and Phase II.

**PHQ-9 Baseline**			**GAD-7 Baseline**		
Diagnostic category cut-off points[a]	Phase I N = 8 n (%)	Phase II N = 16 n(%)	Diagnostic category cut-off points[b]	Phase I N = 8 n (%)	Phase II N = 16 n(%)
None (0-4)	3(38)	7(44)	None (0-4)	4(50)	11(69)
Mild (5-9)	3(38)	8(50)	Mild (5-9)[c]	3(38)	4(25)
Moderate (10-14)	1(13)	1(6)	Moderate (10-14)	1(13)	1(6)
Moderately Severe (15-19)	1(13)	-	Severe (15+)	-	-
Severe (20-17)	-	-			

[a]Kreonke & Spitzer 2002
[b]Spitzer et al 2006
Caseness PHQ-9 score ≥10
Caseness GAD-7 score ≥8
[c]GAD-7 Caseness falls in middle of Mild range. Individual scores in Mild range: Phase I – 5,7,9. Phase II – 6,6,7,6.
