# Supplementary material for: A Web-Based Intervention to Reduce Distress After Prostate Cancer Treatment: Development and Feasibility of the Getting Down to Coping Program in Two Different Clinical Settings
Source: JMIR Cancer. 2018 Apr 30;4(1):e8. doi: 10.2196/cancer.8918 (PMC5952123; doi:10.2196/cancer.8918)
Supplement: Multimedia Appendix 7 [file cancer_v4i1e8_app7.pdf]

Multimedia Appendix 7.

Self-Efficacy change scores in Phase II.

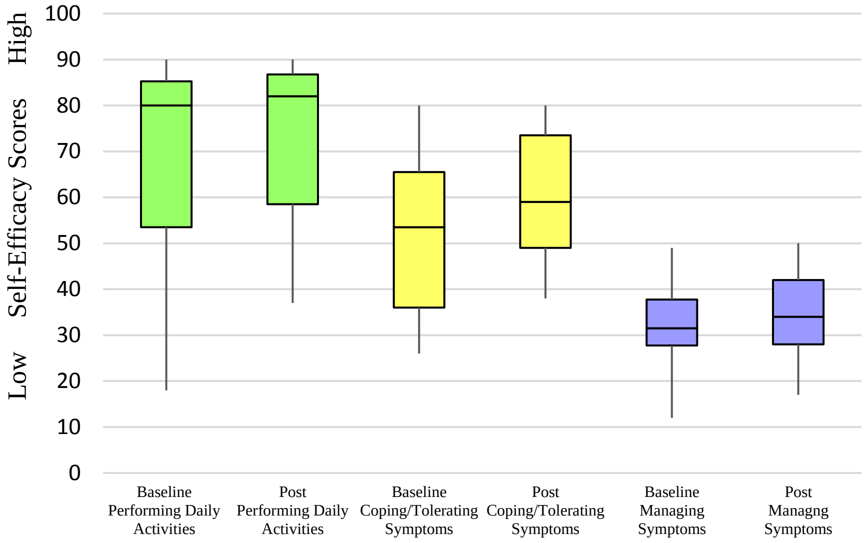

Prostate Cancer Self-Efficacy Scores n = 16
