# Supplementary material for: A Web-Based Intervention to Reduce Distress After Prostate Cancer Treatment: Development and Feasibility of the Getting Down to Coping Program in Two Different Clinical Settings
Source: JMIR Cancer. 2018 Apr 30;4(1):e8. doi: 10.2196/cancer.8918 (PMC5952123; doi:10.2196/cancer.8918)
Supplement: Multimedia Appendix 8 [file cancer_v4i1e8_app8.pdf]

Multimedia Appendix 8.
Phase I and Phase II facilitator verbatims.

About Supporting people with long-term conditions

"I guess one thing I've learnt by working with people that present with long term conditions is that in terms of what we're seeing in their scores, you often don't see much of a change, because it's their physical health condition that's triggering some of their responses… That can be very difficult for people to separate [...]" (Psychological Practitioner 1)

About Ease of engagement and access

"[There] was a lot of the material that was quite simplistic. I mean, what we do is quite simplistic, to a certain extent, but I wasn't sure how [the men] would take to it. […] but I think they took loads from it, actually. And I think it needed to be simplistic because, particularly if you're not in a great place and you're having to […] log on and go through the stats, you could potentially feel a bit overwhelmed. It could be tempting to not continue with it […] but it did seem, you know, just thinking about goals seemed like it was quite powerful for people." (Psychological Practitioner 1)

"[…] because it was more simplistic, it meant that, you know, they obviously needed a lot of self-motivation to want to login to that and work through it, because it's not like having an appointment with us, where we're calling them." (Psychological Practitioner 2)

"[…] we can make assumptions sometimes with healthcare professionals and I think we also focus too much sometimes on the physical and we don't necessarily, for a variety of reasons, whether it's skills, whether it's time, whether it's confidence or you know the misconception that we don't have therapies locally, that we perhaps don't maybe explore the emotional and the psychological issues for people." (Nurse)

About the Facilitator role

"I didn't really feel like I was using my skills […]. I guess partly, that's because the engagement in the [chat room] wasn't massive. If there had been more kind of questions that had come up then maybe that would've been different. […] a lot of the comments that came up in my [chat room] were more statements, rather than asking anything. And it's hard to kind of put a therapy hat on and try and reply to a statement, when they're not actually asking you for anything. I just felt like I was, just going in and checking to see if there were any questions and kind of hoping that there was something to, to support them along with." (Psychological Practitioner 2)

"There was never anything directly asked of me at all. It was more people put things out there." (Psychological Practitioner 1)

"I think first and foremost it was the not knowing what people might post […] and then being quite nervous about, you know, being clear about not being a nurse and being a facilitator […] so not wanting to be that 'fixer.' And then, I think, I was excited, I was keen to see how something online would work, but I guess I suppose just from my own lack of experience, you know, […] because it was new and it was something I hadn't done before […]." (Nurse)

"So I think for me it's maybe about developing my facilitation skills …I watch people that are facilitators and it is an art and it is a skill …my comfort zone is probably face-to-face, one-to-one, […] I think, it's just about building up your confidence and your skills around facilitation and being mindful of the language you use […] and not always trying to fix it, and […] turning it round to people and saying to people "what do you think might help?" […fixing] rather than intervention, rather than […] telling them what to do …" (Nurse)

About Asynchronous chat

"That's what I think would've been helpful. Rather than waiting two days, three days to reply to something, to have been able to reply, maybe not immediately, but on the day, in order to kind of keep the dialogue going. Because it was very much someone says this and then I might say, a few days later "Oh thanks for saying that, what do you think about this?" and then they wouldn't talk again." (Psychological Practitioner 1)

"I can appreciate you're trying to make it practical, why you have the different weeks and why you couldn't access week one, when it was week two. But, it felt like a bit of a shame for the chat room to go. Because some people would write comments on a Saturday or Sunday which could've been great and then, it's Monday, so it's all kind of been taken away. It would've been great to have a rolling chat room." (Psychological Practitioner 2)

"It just seemed that there were things that were kind of getting lost just because it was week three and [the chat room posts] had all gone." (Psychological Practitioner 1)
